# Increased X-ray Visualization of Shape Memory Polymer Foams by Chemical Incorporation of Iodine Motifs

**DOI:** 10.3390/polym9080381

**Published:** 2017-08-20

**Authors:** Landon D. Nash, Mary Beth Browning Monroe, Yong-Hong Ding, Kendal P. Ezell, Anthony J. Boyle, Ramanathan Kadirvel, David F. Kallmes, Duncan J. Maitland

**Affiliations:** 1 Biomedical Engineering, Texas A&M University, College Station, TX 77843, USA; nashlandon@gmail.com (L.D.N.); mbbmonroe@tamu.edu (M.B.B.M.); kendal.ezell@gmail.com (K.P.E.); tony.boyle2011@gmail.com (A.J.B.); 2 Department of Radiology, Mayo Clinic, Rochester, MN 55905, USA; Ding.YongHong@mayo.edu (Y.-H.D.); Kadir@mayo.edu (R.K.); kallmes.david@mayo.edu (D.F.K.)

**Keywords:** shape memory polymer, polymer foam, medical device

## Abstract

Shape memory polymers can be programmed into a secondary geometry and recovered to their primary geometry with the application of a controlled stimulus. Porous shape memory polymer foam scaffolds that respond to body temperature show particular promise for embolic medical applications. A limitation for the minimally invasive delivery of these materials is an inherent lack of X-ray contrast. In this work, a triiodobenzene containing a monomer was incorporated into a shape memory polymer foam material system to chemically impart X-ray visibility and increase material toughness. Composition and process changes enabled further control over material density and thermomechanical properties. The proposed material system demonstrates a wide range of tailorable functional properties for the design of embolic medical devices, including X-ray visibility, expansion rate, and porosity. Enhanced visualization of these materials can improve the acute performance of medical devices used to treat vascular malformations, and the material porosity provides a healing scaffold for durable occlusion.

## 1. Introduction

Shape memory polymers (SMPs) show significant promise for use in a variety of medical applications, including programmed surfaces for controlled cellular differentiation and alignment, microactuators, and implanted medical devices [1,2,3,4]. Specifically, thermally actuated ultralow density SMP polyurethanes synthesized as expanded, open porous foams have shown promise for endovascular occlusion of diseased peripheral blood vessels and intracranial aneurysms [5,6,7,8]. These vascular malformations contribute to complications such as severe chronic venous insufficiency, and subarachnoid hemorrhage [9,10]. SMP foams can improve upon the current standard of care for vascular occlusion, endovascular coils, by enhancing clotting and healing while maintaining minimally invasive delivery. These programmable materials can be heated, crimped, and cooled into a metastable secondary geometry. This low-profile geometry allows for catheter delivery to defect sites, upon which the foam is stimulated by exposure to body temperature blood to recover to its expanded primary geometry [11]. Utilization of SMP foams in conjunction with current coiling techniques can reduce the number of coils necessary to fill an intracranial saccular aneurysm to decrease procedure time and cost [7]. SMP foam expansion forces are significantly lower than those applied by bare platinum coils (BPCs), lowering the risk of aneurysm dissection during implantation [12]. The embolic foam occludes the aneurysm by disrupting blood flow and inducing clot formation within the foam pores. Once a clot has formed, the foam serves as a scaffold to promote collagenous scar formation for durable aneurysm occlusion in animal models [13,14].

**Figure 1:**
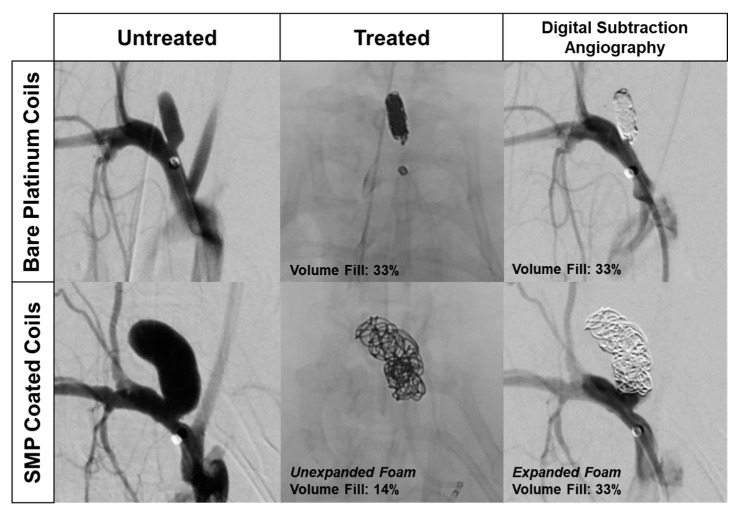
Rabbit elastase aneurysm treated with (**Top Row**) bare platinum coils (BPCs) and (**Bottom Row**) shape memory polymer (SMP) foam coated coils. Middle Column—BPCs have a more dense 2D radiographic projection during device delivery. Right Column—Calculated volumetric occlusion for both aneurysms is the same.

Delivery of SMPs into aneurysms and peripheral vasculature requires X-ray imaging. X-ray visualization is essential for proper and safe device placement into the target anatomy. In the case of cerebral aneurysm embolization, the lack of SMP X-ray contrast can be partially addressed by utilizing the SMPs as a coating over a radiodense BPC [7]. However, this approach is limited, as highlighted in Figure 1. The left column depicts the typical anatomy of a rabbit elastase aneurysm model. The rabbit elastase model is a common model for assessing the safety and efficacy of implantable neurovascular medical devices. The SMP foam device implantation data shown in Figure 1 followed previously published aneurysm model creation methods and standard endovascular procedures [15,16,17,18]. The top row shows angiography of an aneurysm treated with traditional BPCs, depicting the dense 2D projection of the coil mass that clinicians use as the primary indicator of sufficient aneurysm packing. Although the aneurysm appears to be densely packed with coils, the average packing density of embolic coils ranges between 30–35% [19]. The bottom row shows an aneurysm treated with SMP foam-coated embolic coils [7]. The X-ray image in the middle column indicates that the aneurysm is loosely filled, but the limited ingress of the injected contrast agent in black (right column) proves that the interstitial spaces between the coils are filled with non-X-ray visible embolic foam and thrombus. While injected contrast agent can serve as an indicator of aneurysm embolization, it deviates from the standard 2D X-ray image technique, and therefore presents a hurdle for clinical adoption of SMP foam devices. If standard 2D X-ray imaging was utilized with foam-coated coils, it could potentially lead to over-packing of the aneurysm. X-ray visible SMP foams could reduce the risk of these complications and improve clinician visualization of true volumetric occlusion.

Previously, polymer systems have been made X-ray visible through the physical incorporation of tantalum particles, inorganic nanoparticles, and custom iodine containing contrast agents [20,21,22]. Specific to SMP foams, X-ray visibility has been increased through the incorporation of tungsten microparticles into the foam matrix [23]. However, this approach results in composites with diminished toughness, which raises concerns over particulate generation in vivo and subsequent emboli in the blood stream. Furthermore, the degree of opacification achieved with microparticle incorporation is not sufficient for small diameter, low-density devices, such as those used for neurovascular aneurysm embolization. Radiopaque nanoparticulate additives were investigated as an alternative to address decreases in material toughness by increasing dispersion within the matrix. Low concentrations of nanoparticulates improved mechanical strength and toughness, but increasing nanoparticulate concentration to that needed for sufficient X-ray visualization diminished foam mechanical properties [24,25]. Thus, there is a need for X-ray visible SMP foams with maintained mechanical integrity.

As an alternative to radiopaque micro or nanoparticles, this work focuses on the chemical incorporation of iodine motifs into the polymer matrix to increase X-ray visibility without affecting bulk foam properties. The most typical iodine functional group used in biomedical applications is the triiodobenzene ring. This functional group is used in all commercial injectable X-ray contrast agents to achieve iodine solution concentrations in the range of 150–300 mg of iodine per milliliter (mg I/mL) [26,27].

When compared to particulate additives, the chemical approach of incorporating triidobenzene monomers into the material during synthesis was hypothesized to enable higher contrast loading, without affecting the mechanical integrity of the bulk material. This radiodense SMP system can be utilized to create low density foams for embolic applications without the need for metal components, such as platinum backbones (e.g., BPCs) or marker bands. This material system could set the stage for entirely polymeric, degradable devices for use in a variety of applications in addition to cerebrovascular and peripheral embolization. Furthermore, while this work focuses on the incorporation of iodine into a specific SMP foam system, the information gained here could be applied to a range of polymeric biomaterials to enable imaging during implantation.

**Figure 2:**
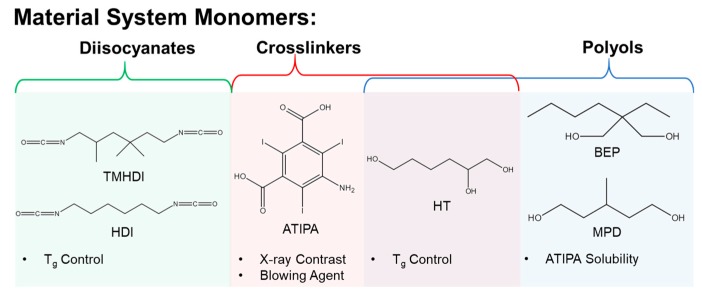
Investigated monomers for X-ray visible SMP development.

Figure 2 depicts the monomers selected to investigate an X-ray visible SMP system. Aliphatic isocyanates were chosen for a polyurethane composition based on previous SMP materials with favorable biocompatibility in embolic applications [13,14]. Changing the molar ratios of hexamethylene diisocyanate (HDI) and trimethylhexamethylene diisocyanate (TMHDI) was hypothesized to enable control over bulk material hydrophobicity and glass transition temperature (T_g_), for a tailored material expansion rate [28]. The proposed contrast agent monomer is 5-amino-2,4,6-triiodoisophthalic acid (ATIPA). The X-ray contrast of the ATIPA molecule is derived from the triiodobenzene motif, which incorporates three high-z iodine atoms. ATIPA is terminated with a primary aromatic amine and two carboxylic acids, giving it a functionality of three for crosslinking reactions with isocyanates. Further, the reaction between isocyanates and carboxylic acids yields a carbon dioxide byproduct, making ATIPA a chemical blowing agent during foam polymerization [29]. Solubility was a significant barrier for the development of this system. ATIPA is a hydrophilic solid monomer with negligible solubility in the chosen aliphatic diisocyanates. While ATIPA is soluble in tetrahydrofuran and dimethylsulfoxide, a solvent-free synthetic procedure is preferable to mitigate organic contaminants in end-use medical products. The proposed polyols, 2-butyl-2-ethyl propanediol (BEP), 3-methyl-1,5-pentanediol (MPD), and 1,2,6-hexanetriol (HT) were selected based on favorable ATIPA solubility and T_g_ control in the final material.

## 2. Materials and Methods 

### 2.1. Foam Synthesis

Table 1 summarizes all of the foam compositions that were synthesized. For the non-isocyanate portion of the polyurethane, the reactive hydroxyl, amine, and carboxylic acid functional groups were considered for the synthesis calculations. A 2% molar excess of isocyanate was added to each foam during synthesis to account for ambient moisture contamination during foam mixing. HDI, TMHDI, BEP, MPD, ATIPA, and HT were used as received from VWR Scientific (Radnor, PA, USA) and Sigma Aldrich (St. Louis, MO, USA). The physical blowing agent, Enovate 245fa (Eno), was used as received from Honeywell. 

For foaming, hydroxyl (OH) premixes were prepared 1 day prior to foaming by combining a 0.6 equivalent ratio of non-isocyanate monomers (ATIPA, BEP, MPD, HT) into a 15 mL polypropylene Flacktek mixing cup. The contents were mixed for 30 s at 3400 rpm in a Flacktek high speed shear mixer, heated at 50 °C for 1 h, mixed again for 30 s at 3400 rpm, and heated overnight at 50 °C. Anhydrous tetrahydrofuran (THF) was added at 5 wt % to the OH premix to aid in solubility for the 30 eq % ATIPA composition.

Viscous isocyanate (NCO) premixes were prepared in a desiccated glovebox by adding a 0.4 molar ratio of reactive non-isocyanate equivalents to the entirety of the diisocyanate equivalents in a 150 mL polypropylene Flacktek mixing cup. The contents were mixed at 3400 rpm for ten minutes until a single phase was achieved. This premix was shaken at 1 rpm at room temperature for 2–5 h until the mixture achieved a room temperature viscosity comparable to that of honey. For the 0AT composition, 0.04 g of DABCO T131 gelling catalyst and 0.08 g of DABCO BL22 blowing catalyst were added to increase the reaction rate during foaming.

To stabilize the foam during blowing, 4 wt % DCI990 surfactant (DABCO) was added to the NCO premix and mixed for 30 s. The OH premix was added to the NCO premix and mixed for 30 s. Approximately 1–2 mL of Eno was immediately added to the reactive resin and mixed for 30 s. The reaction was quickly moved to a 90 °C oven for a 20 min cure. After curing, the foam skin was removed with a razor, and the foam was post-cured at 50 °C for 12 h. Post-cured foams were cubed and stored with desiccant in polypropylene bags.

### 2.2. Physical Characterization

Six cubes measuring approximately 1 cm^3^ were taken from each composition for density measurements. Density was calculated as the sample mass divided by the product of the length, width, and height of the sample (volume).

Pore sizes were determined from light microscopy images acquired at 50× and 100× magnification using a Keyence VHX-5000 metrology system (Keyence, Osaka, Japan) with a variable illumination adapter. Foam samples were cut into 2–4 mm slices in the axial and transverse planes. Ten pore diameter measurements were taken from each foam image.

### 2.3. Gel Fraction

Foam samples measuring approximately 1 cm^3^ were cleaned to remove residual surfactant using three 30 min sonication intervals in isopropyl alcohol at a 20:1 volume dilution ratio. The samples were dried under vacuum at 100 °C for 12 h. Dried foam samples were massed and added to 20 mL vials filled to the shoulder with THF, and heated at 50 °C with 1 Hz oscillation for 48 h. The THF was removed and samples were dried under vacuum at 70 °C for 36 h. Gel fraction is reported as the final sample mass divided by the original sample mass.

### 2.4. Differential Scanning Calorimetry (DSC)

Dry T_g_ was determined using a TA Q200 Differential Scanning Calorimeter (TA Instruments, New Castle, DE, USA) on 5–10 mg foam samples in a vented aluminum pan. The samples were equilibrated at −40 °C for 5 min, heated to 120 °C, cooled to −40 °C, and reheated to 120 °C at temperature ramps of 10 °C/min. T_g_ was calculated as the inflection point of the second heating curve. 

Wet T_g_ foam samples were immersed in 50 °C water for 30 min to achieve moisture plasticization. Excess moisture was removed by compressing the foam between tissue paper at 2 tons for 30 s using a Carver laboratory press. Foam samples (5–10 mg) were added to an aluminum pan, which was hermetically sealed. Samples were cooled to −40 °C, equilibrated for 5 min, and heated to 100 °C at 10 °C/min. Wet T_g_ was calculated from the heating curve inflection point.

### 2.5. ATR FTIR

**Figure 3:**
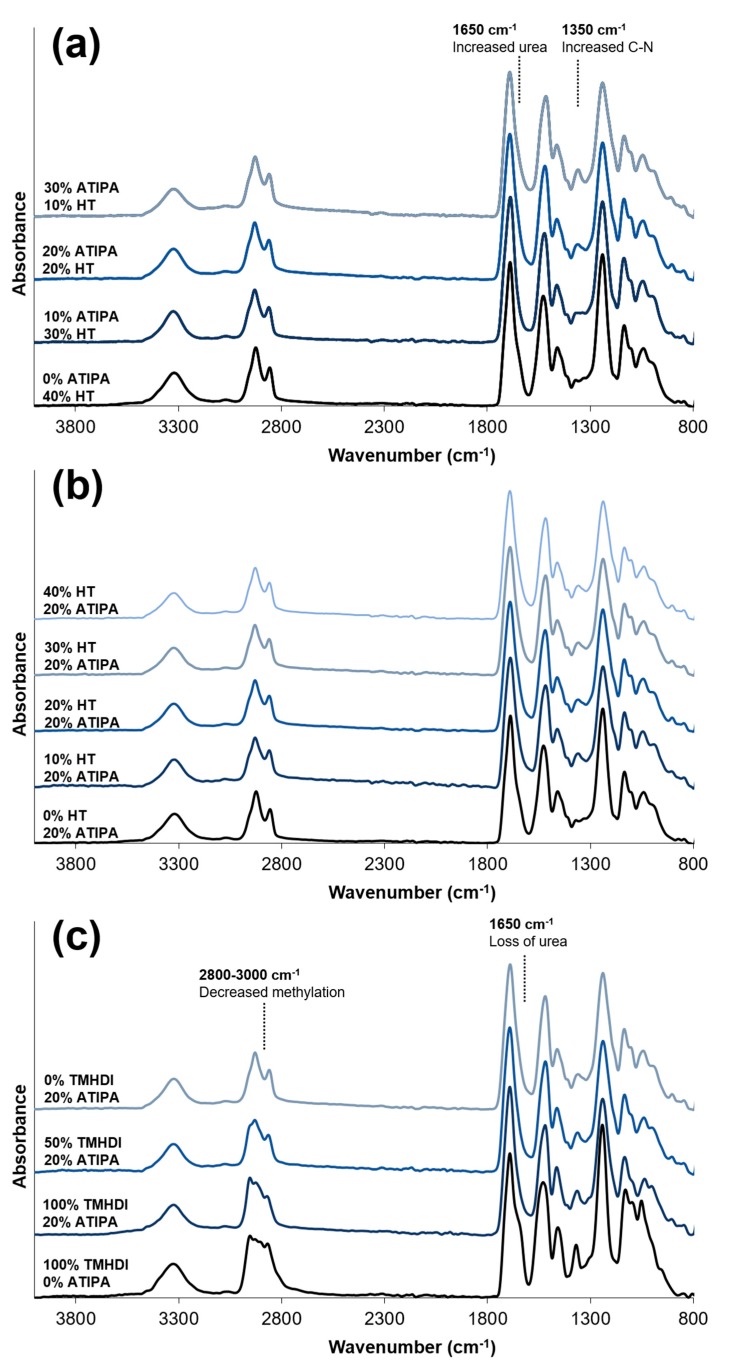
Attenuated Total Reflectance Fourier Transform Infrared (ATR FTIR) spectra for foams with varying (**a**) 5-amino-2,4,6-triiodoisophthalic acid (ATIPA) content; (**b**) 1,2,6-hexanetriol (HT) content; and (**c**) isocyanate content.

ATR FTIR spectra were obtained using a Bruker ALPHA Infrared Spectrometer (Bruker, Billerica, MA, USA) with a diamond ATR crystal. Data analysis was conducted using Bruker OPUS Spectroscopy software (Bruker, Billerica, MA, USA; 2016). FTIR spectra of the Enovate foam series are not included in Figure 3, because the physical blowing agent does not alter the foam chemistry.

### 2.6. X-ray Imaging

Foam samples were prepared for X-ray imaging by cutting 1 cm × 1 cm samples into 8, 4, 2, and 1 mm thick slices, and adhering them to a polypropylene sheet. Each foam array included a platinum embolic coil (GDC^®^ 360° Detachable Coil (Stryker, Kalamazoo, MI, USA) or 0.008′′ OD 92/8 Pt/W coil) as an X-ray visualization standard. Peripheral occlusion prototypes were prepared by cutting the foams into 8 mm diameter cylinders, and axially threading them over a 0.006′′ stainless steel wire. One prototype was radially crimped using a Machine Solutions SC250 heated stent crimper (Machine Solutions, Flagstaff, AZ, USA). The sample was equilibrated at 100 °C in the crimping bore for 15 min, radially compressed, and constrained while cooling to an ambient temperature. Crimped and expanded peripheral occlusion prototypes were imaged. Neurovascular prototypes (2 mm in diameter) were also prepared without a backbone wire and radially crimped. X-ray images were acquired using a Philips Allura Xper FD20 X-ray system (Philips, Amsterdam, The Netherlands) in angiography mode. Samples were imaged directly and through a 0.5′′ aluminum plate, used as an X-ray attenuating skull analog [30].

Average 8-bit decimal grayscale values for each fluoroscopy image sample were analyzed using ImageJ. These sample values were reported as a grayscale value shift relative to the image background grayscale value.

### 2.7. Dynamic Mechanical Analysis (DMA)

Dynamic mechanical analysis was conducted using a TA Q800 Dynamic Mechanical Analyzer (TA Instruments, New Castle, DE, USA) in compression mode. Foam cylinders were prepared using an 8 mm biopsy punch, and cut to approximately 5 mm in length using a razor. Samples were equilibrated to 0 °C for 5 min, and heated to 120 °C at 3 °C/min while undergoing 40 μm deformations at 1 Hz.

### 2.8. Unconstrained Expansion

Foams with varying HT content were cut into 2 mm diameter cylinders and axially threaded over 0.006′′ stainless steel wires. Samples were radially compressed using a Machine Solutions SC250 heated stent crimper. Crimped samples were allowed to relax for 24 h before being expanded in a 37 °C water bath. Samples were imaged at 5 min intervals for a total of 45 min. Five diameter measurements were taken along the length of the expanding foam using ImageJ software.

### 2.9. Tensile Testing

Dry foam samples were prepared using an ASTM Type IV dog bone punch. Uniaxial tensile tests were conducted at room temperature using an Insight 30 Material Tester (MTS Systems Corporation, Eden Prairie, MN, USA) with a constant strain rate of 50 mm/min. Ultimate tensile strength (kPa), strain at break (%), and elastic modulus (kPa) were calculated from the stress-strain curve of each sample (*n* = 8). Toughness was calculated as the area under the stress-strain curve using the trapezoidal rule.

### 2.10. Statistical Analysis 

Dry T_g_ values for each composition series were compared using a 1 way ANOVA test (α = 0.05).

## 3. Results and Discussion

Physical and thermomechanical properties for all foam compositions are summarized in Table 2. Each foam series is grouped together and named after the variable altered in the synthetic procedure. Average gel fractions for the selected compositions ranged between 94.5–99.0%. These values are comparable to those reported in previous non-radiopaque SMP foam formulations [6]. High gel fractions reduce the risk for complications related to leachable chemicals exiting a permanently implanted biomaterial. If necessary, this risk could be further mitigated with a more rigorous foam cleaning protocol to remove any potential unreacted leachables prior to device implantation.

After characterizing foams 1–16, compositions 17 and 18 were fabricated at a 4× scale for tensile testing and prototype fabrication. These compositions incorporated chemistry changes to achieve desirable morphology and thermomechanical properties.

### 3.1. Effects of Varying ATIPA Content

The ATIPA content was varied in compositions 1–4. HT content was simultaneously changed to maintain a consistent theoretical crosslink density based upon isocyanate-reactive functional groups. Bulk density decreased with increasing ATIPA content, due to the blowing reaction generated by the two carboxylic acid groups on the ATIPA monomer. Dry T_g_ increased with increasing ATIPA content. Although the theoretical crosslink density for these compositions is constant, the aromatic structure of ATIPA is more rigid than the aliphatic HT monomer, increasing the network rigidity and glass transition temperatures.

FTIR spectra of compositions with increasing ATIPA content, Figure 3a, show a broadening urea shoulder at 1650 cm^−1^ due to the reaction between NH_2_ reactive groups on ATIPA and isocyanates during synthesis. These spectra provide verification of the chemical incorporation of ATIPA into the polyurethane SMP foams. 

**Figure 4:**
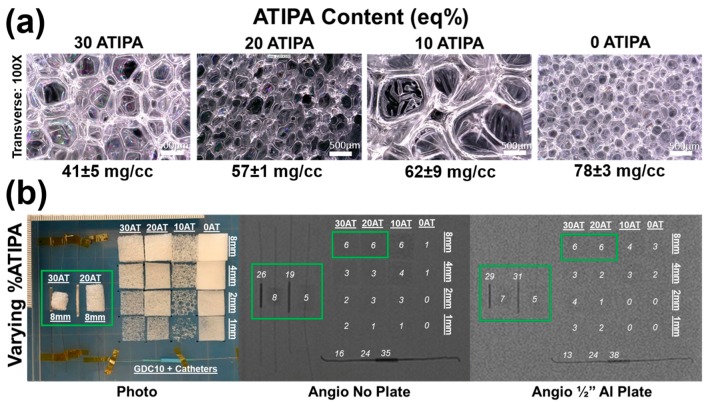
(**a**) Light microscopy images of foams with varying ATIPA content at 100× magnification. Images are labeled with average ± standard deviation of density measurements (*n* = 6); (**b**) X-ray images of foams with varying ATIPA content at varying thicknesses. 8 mm diameter peripheral embolization prototypes are boxed in green. Commercial platinum embolic coil, microcatheter, and guide catheter segments are included for comparison. Each sample is labeled with the average 8-bit decimal grayscale shift relative to the image background.

Images of the foams synthesized with varied ATIPA content (0–30 eq %) are shown in Figure 4a. These foams have significant differences in pore morphology because the foaming parameters (premix non-isocyanate ratio, premix cure time, premix viscosity, etc.) were not altered to compensate for changes in ATIPA content. It is important to note that the 30 eq % ATIPA composition required 5 wt % of anhydrous THF during synthesis to prevent ATIPA precipitation.

Figure 4b summarizes the X-ray visibility of low-density foams with varying ATIPA content. The foams with 10–30 eq % ATIPA demonstrate comparable X-ray visibility, because the chemical blowing reaction between ATIPA and HDI resulted in lower material density at higher contrast agent loading (Table 2). These opposing trends resulted in comparable iodine content within the same expanded sample path length. The 8 mm cylindrical peripheral embolization prototypes demonstrated visibility in their expanded state, even when imaged through the skull analog (½′′ Al plate). When radially crimped, these materials demonstrated visibility comparable to commercially available embolic platinum coils with grayscale shifts between 24 and 30. This quantitative comparison suggests that these prototype compositions and dimensions are appropriate for delivery and visualization in peripheral tissues.

Radial compression compensates for differences in bulk material density, revealing differences in X-ray visualization for the samples with varying ATIPA content. For example, the radially compressed 30AT sample is noticeably more visible than the 20AT sample when imaged through the ½′′ Al skull analog. These results indicate that foam density has a large effect on radiopacity and that increased ATIPA concentration can be used to enhance X-ray visibility. Based on the favorable radiopacity and the lack of need for solvent in foaming, the 20% ATIPA formulation was selected for all further studies of material variables.

### 3.2. Effects of Varying Foam Density

The physical blowing agent, Enovate, was utilized to tune ATIPA-containing foam density. For compositions 5–9 with varying volumes of Enovate, there is a clear trend of decreasing material density with increasing volume of Enovate (Table 2). Although there is a statistically significant difference in the dry Tg values, this variation is not expected to significantly affect device design criteria. 

**Figure 5:**
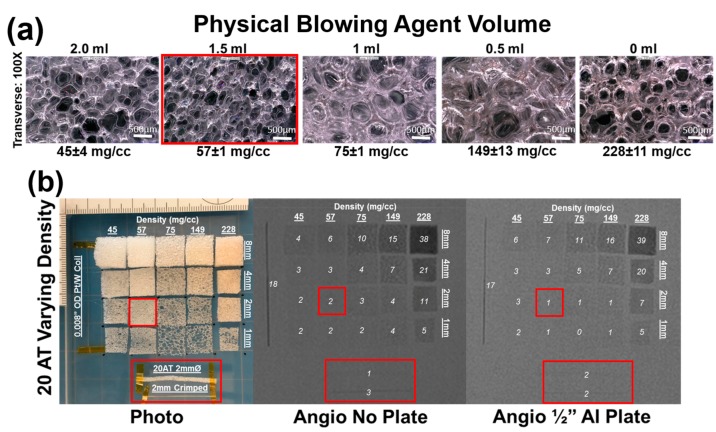
(**a**) Light microscopy images of foams with varying density at 100× magnification. Images are labeled with average ± standard deviation of density measurements (*n* = 6); (**b**) Fluoroscopy images of 20% ATIPA foams with varying density at varying thicknesses. Neurovascular device prototypes and their corresponding foam formulation are boxed in red. A 92/8 Pt/W coil with 0.008′′ OD is included for comparison. Each sample is labeled with the average 8-bit decimal grayscale shift relative to the image background.

Figure 5a illustrates the impact of physical blowing agent volume on foam morphology. The “0 mL” composition that uses no physical blowing agent still has porosity due to the ATIPA blowing reaction. Although these pores are comparable in size to the 0.5 mL and 1.0 mL Enovate foams, the thick strut morphology contributes to the significant difference in bulk material density. Similarly, the smaller pores in the 1.5 and 2.0 mL foam yield the least dense materials due to thin struts.

Figure 5b details foams with a fixed 20 eq % ATIPA composition at varying density (i.e., Enovate volume). Qualitative assessment suggests a grayscale shift of 5 is required for minimum visibility. Increasing material density results in increased material visualization, with the densest foam exhibiting visibility at 1 mm thickness, even through the skull analog. However, the 2 mm neurovascular prototypes made with low-density foams displayed a limited visibility, even when axially crimped. To address the limited material visibility at the neurovascular device scale, future prototypes could incorporate foams with higher ATIPA percentages. Alternatively, it is hypothesized that a combinatory approach of chemical opacification and radiodense nanoparticulate loading could achieve SMP foam composite visualization at neurovascular device scales. For appropriate clinical performance, future prototypes should have a minimum optical density contrast of 0.05 to comply with recommendations of radiopacity standards [31,32].

Visualization of the expanded material would be ideal for clinical adoption without extensive physician training. However, visible crimped devices would also represent an improvement in device performance. Devices would be visible during delivery, and instead of direct visualization of foam expansion, the gradual decrease in material visualization would provide a secondary indication of foam expansion.

### 3.3. Effects of Varying Hexanetriol Content

Compositions 10–13 with varying HT content have comparable density and consistent average pore diameters in the 300–400 μm range (Table 2). There is a statistically significant difference between the dry T_g_’s for each composition. The gel fraction data indicates that the increasing HT content increases the material crosslink density. The higher amount of tri-functional polyols relative to diols effectively decreases the molecular weight between crosslinks to increase network rigidity and T_g_. Compositions 10–13 demonstrate that HT concentration is an effective way to control T_g_ at fixed contrast agent loading (20% ATIPA) and foam density.

Figure 3b shows that the FTIR spectra do not significantly change with increasing HT content. However, these spectra do show peaks characteristic of polyurethane foams [33,34,35]. The broad peak centered at 3310 cm^−1^ highlights the N-H vibrations. Peaks at 2852 and 2923 cm^−1^ are from symmetric and asymmetric C-H stretching from the MPD methyl group. The lack of an unreacted NCO peak at 2260 cm^−1^ should be noted. At 1685 cm^−1^, the urethane C=O peak is shifted to the right due to hydrogen bonding. This is congruent with hydrogen bonded urethane peaks in other polyurethane SMP foams with relatively low molecular weights between crosslinks when compared to segmented polyurethanes [6]. Additionally, strong amide II and amide III peaks can be seen at 1515 and 1230 cm^−1^, respectively.

**Figure 6:**
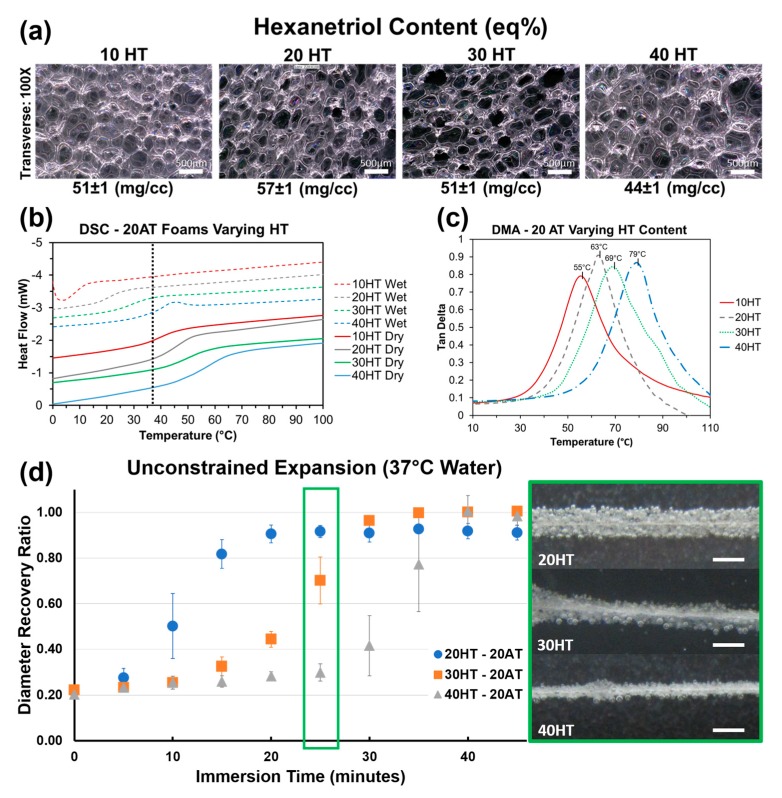
(**a**) Light microscopy images of 20% ATIPA foams with varying HT content at fixed density. (**b**) Differential scanning calorimetry (DSC) thermograms for the same foam series under wet and dry conditions. The dotted vertical line at 37 °C represents body temperature; (**c**) Compression DMA for the same foam series; (**d**) Unconstrained foam expansion profiles in a body temperature (37 °C) water bath. Scale bars are 1 mm.

Figure 6a depicts foams with comparable pore size, density, and strut morphology with varying HT content. It is important to note that composition, and subsequent thermomechanical properties can be altered independently of foam morphology to enable material optimization towards target medical device applications. Using both chemical blowing with ATIPA and physical blowing with Enovate enables morphology control of SMP foams.

Figure 6b shows incremental control over both dry and moisture plasticized T_g_ based on HT composition. Increasing the molar ratio of the trifunctional HT monomer increases the crosslink density to make the polymer structure more rigid and raise the T_g_. For each composition, the wet and dry transitions are positioned on either side of body temperature (37 °C), enabling passive material expansion once implanted in the body. Both the transition temperature value and the breadth of transition increase with increasing HT content. Based on these thermograms, the 20HT, 30HT, and 40HT compositions were chosen for further analysis towards device development. The 10HT composition was determined to have a dry transition too low for sufficient working times at the neurovascular device prototype scale.

DMA analysis for foams with varying HT content is summarized in Figure 6c. Peak tan δ values are approximately 20 °C higher than DSC dry T_g_ values in Figure 6b. However, the incremental differences in each composition are comparable. This DMA data further supports increasing T_g_ with increasing HT content.

Foams with varying HT content displayed volumetric recovery behavior in body temperature water that is congruent with the T_g_ trends. As seen in Figure 6d, compositions with a higher HT content and a higher T_g_ took longer to expand. The 30 and 40 eq % HT compositions also showed higher average volumetric recovery (99%) when compared to 20 eq % HT foams (91%). This result is attributed to the lower crosslink density of the 20 eq % HT foam. This data shows promise for neurovascular embolization device design. With an average crimped diameter of 0.0196 ± 0.001′′, these prototypes can be improved for 0.021′′ microcatheter delivery with a target minimum tolerance of 0.002′′. Modifications to the blowing agent and surfactant composition can decrease the bulk foam density for smaller crimped dimensions. The expansion profiles show an estimated minimum working time of 10 min for the 30HT composition and 15 min for the 40HT composition.

### 3.4. Effects of Varying Isocyanate Content

Compositions 14–16 all have 20 eq % ATIPA with varying isocyanate content. Changing the isocyanate composition demonstrated a less significant effect on T_g_ than altered HT content. FTIR spectra of 20 eq % ATIPA foams with increasing TMHDI content are shown in Figure 3c. Non-radiopaque 100% TMHDI foam is included for comparison. Increased methylation with increasing TMHDI content is evident due to peak broadening between 2800–3000 cm^−1^. Compared to the non-radiopaque foam, the ATIPA foams have a reduced urea shoulder at 1650 cm^−1^. In traditional polyurethane foaming, water is used as a blowing agent, which produces urea linkages in the backbone. The carboxylic acid groups in ATIPA provide an alternate chemical blowing agent, and allow for foam synthesis without water. The lack of water in the ATIPA foam synthesis results in fewer urea linkages relative to traditional non-radiopaque foams made with water. Although foams with increased TMHDI content were successfully fabricated, they were qualitatively less tough when compared to HDI foams, and were not selected for further device optimization.

### 3.5. Mechanical Properties

**Figure 7:**
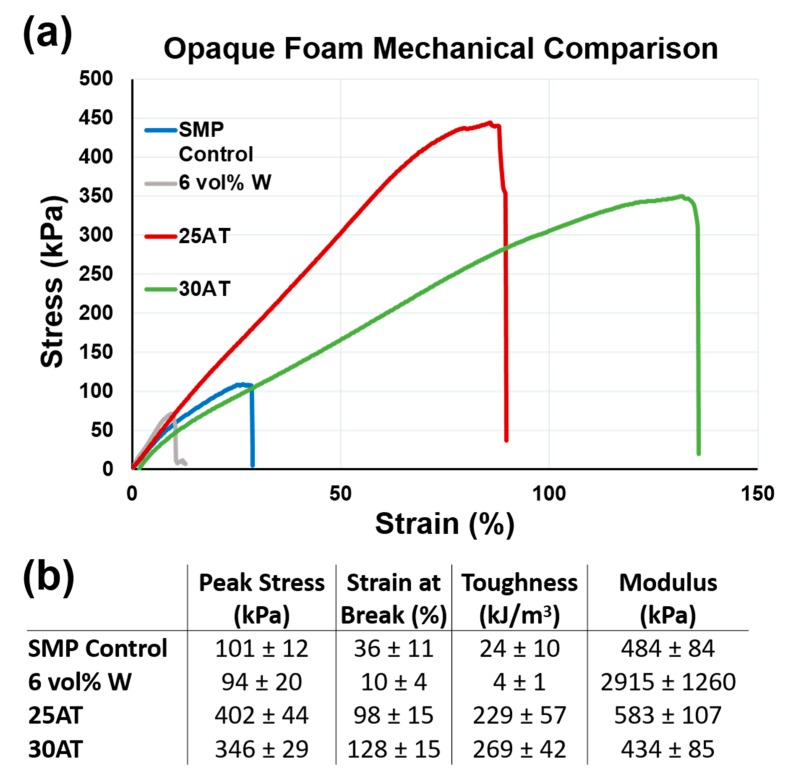
(**a**) Representative stress-strain curves for X-ray visible foams (ATIPA and tungsten loaded) and non-visible control foam; (**b**) Mechanical properties (mean ± standard deviation).

Figure 7 shows representative uniaxial tension stress vs. strain curves and affiliated calculations for a non-visible SMP foam, a 6 vol % tungsten nanoparticulate-loaded SMP composite (6 vol % W), a 25 eq % ATIPA foam, and a 30 eq % ATIPA foam (compositions 17 and 18) [18,24].

ATIPA foam compositions have significantly higher toughness when compared to previous low-density SMP foams. The 25AT and 30AT compositions had average peak stress values of 402 ± 44 kPa and 346 ± 29 kPa, respectively. The increased material strength (peak stress) is attributed to the aromatic structure of the ATIPA monomer. Aromatic compounds traditionally display higher strength when compared to their aliphatic counterparts [36]. Although this polymer system was designed to be biodurable with minimal sites for oxidative or hydrolytic degradation, the biocompatibility of potential aromatic degradation products will be investigated in future studies to ensure the safety of radiopaque SMP foams. 

The 25AT and 30AT compositions had average strain at break values of 98 ± 15% and 128 ± 15%, respectively. Increases in ductility are attributed to a decrease in crosslink density when compared to the non-visible control SMP foam. The non-visible SMP control uses polyols with functionalities of three (triethanolamine) or four (*N*,*N*,*N*′,*N*′-tetrakis (2-hydroxypropyl) ethylenediamine). These crosslinking sites are bridged by short diisocyanate segments (TMHDI or HDI) to create a highly crosslinked material. This crosslink density affords excellent shape memory, but at the relative expense of overall toughness. This reduced toughness is not problematic for a standard foam, but tungsten-loaded composites introduce stress concentrations in the strut cross section to further reduce the overall toughness. The proposed ATIPA compositions employ the aliphatic diols MPD and BEP to increase molecular weight between crosslinks for increased ductility. The rigidity of ATIPA enables chain extension while maintaining the dry transition temperature of the overall material within a functional biomedical range (40–60 °C). The rigidity of the ATIPA structure and diol chain extenders also contribute to the elastic modulus values comparable to the control SMP composition. 

The combined increases in ductility and strength contribute to significant increases in tensile toughness. Compared to non-visible foams, 25AT and 30AT compositions are over 11 and 14 times tougher, respectively. Compared to 6 vol % tungsten nanoparticle foams, 25AT and 30AT foams are 57 and 67 times tougher, respectively. This dramatic increase in toughness lowers the risk of undesired embolic particles detaching from the foam and flowing downstream from the target therapeutic region. 

In future work, ATIPA composites with radiopaque nanoparticulates will be synthesized and characterized. Even with nanoparticulate stress concentrators, these composites are hypothesized to maintain higher fracture toughness than the traditional non-visible SMP foam. This property would maintain the existing acceptable risk level for undesired embolic particulates while affording excellent X-ray visualization during device implantation.

## 4. Conclusions

In this work, chemically modified SMP foams with inherent X-ray visualization were successfully fabricated by the incorporation of the triiodobenzene containing monomer, ATIPA. This material system demonstrated improved toughness as compared to previously reported low density embolic SMP foams. Altering the molar ratios of the other constituent monomers also demonstrated functional changes in these polymer scaffolds that show promise for incorporation into embolic medical devices.

The proposed compositions show potential for peripheral embolization applications in soft tissues with minimal attenuation. However, additional work is necessary to increase the visibility of small cross section neurovascular embolization devices used in the X-ray attenuating environment of the human skull.

## Figures and Tables

**Table 1:** 5-amino-2,4,6-triiodoisophthalic acid (ATIPA) foam compositions; MPD: 3-methyl-1,5-pentanediol, BEP: 2-butyl-2-ethyl propanediol, HT: 1,2,6-hexanetriol, NCO: isocyanate, HDI: hexamethylene diisocyanate; TMHDI: trimethylhexamethylene diisocyanate.

No.	Composition	ATIPA (eq %)	MPD (eq %)	BEP (eq %)	HT (eq %)	NCO	Enovate (mL)	Scale (g)
1	0ATIPA—40HT	0	40	20	40	HDI	1.5	8
2	10ATIPA—30HT	10	40	20	30	HDI	2	8
3	20ATIPA—20HT	20	40	20	20	HDI	1.5	8
4	30ATIPA—10HT ^1^	30	40	20	10	HDI	1	8
5	0.0 mL Eno—20ATIPA	20	40	20	20	HDI	0	8
6	0.5 mL Eno—20ATIPA	20	40	20	20	HDI	0.5	8
7	1.0 mL Eno—20ATIPA	20	40	20	20	HDI	1	8
8	1.5 mL Eno—20ATIPA	20	40	20	20	HDI	1.5	8
9	2.0 mL Eno—20ATIPA	20	40	20	20	HDI	2	8
10	10HT—20ATIPA	20	50	20	10	HDI	1.5	8
11	20HT—20ATIPA	20	40	20	20	HDI	1.5	8
12	30HT—20ATIPA	20	30	20	30	HDI	1.5	8
13	40HT—20ATIPA	20	20	20	40	HDI	1.5	8
14	20ATIPA HDI	20	40	20	20	HDI	1.5	8
15	20ATIPA 50HDI/50TM	20	40	20	20	50HDI/50TMHDI	1.5	8
16	20ATIPA TM	20	20	20	20	TMHDI	1.5	8
17	25ATIPA 32 g	25	40	20	25	HDI	1.5	32
18	30ATIPA 32 g ^1^	30	40	20	20	HDI	1.5	32

^1^ 5 wt % Tetrahydrofuran (total synthesis mass) added to hydroxyl (OH) premix.

**Table 2:** Physical and thermomechanical ATIPA foam properties.

No.	Composition	Density (g/cc)	Axial Pore Size (μm)	Transverse Pore Size (μm)	Dry Tg (°C)	Gel Fraction (%)
		*n* = 6	*n* = 10	*n* = 10	*n* = 3	*n* = 5
1	0ATIPA—40HT	0.078 ± 0.003	231 ± 120	233 ± 123	38 ± 0.1	95.8 ± 3.9
2	10ATIPA—30HT	0.062 ± 0.009	1360 ± 723	1130 ± 399	37 ± 0.4	98.7 ± 0.7
3	20ATIPA—20HT	0.057 ± 0.001	293 ± 102	306 ± 115	45 ± 1.2	95.1 ± 0.6
4	30ATIPA—10HT	0.041 ± 0.005	558 ± 346	579 ± 238	52 ± 1.5	96.4 ± 0.6
5	0.0 mL Eno—20ATIPA	0.228 ± 0.011	617 ± 243	574 ± 277	47 ± 0.5	-
6	0.5 mL Eno—20ATIPA	0.149 ± 0.013	1039 ± 523	845 ± 370	44 ± 2.3	-
7	1.0 mL Eno—20ATIPA	0.075 ± 0.001	856 ± 321	974 ± 502	47 ± 0.4	-
8	1.5 mL Eno—20ATIPA	0.057 ± 0.001	293 ± 102	306 ± 115	45 ± 1.2	-
9	2.0 mL Eno—20ATIPA	0.045 ± 0.004	401 ± 158	332 ± 126	44 ± 0.7	-
10	10HT—20ATIPA	0.051 ± 0.001	402 ± 191	311 ± 112	41 ± 1.0	94.5 ± 2.5
11	20HT—20ATIPA	0.057 ± 0.001	293 ± 102	306 ± 115	45 ± 1.2	95.1 ± 0.6
12	30HT—20ATIPA	0.051 ± 0.001	384 ± 152	364 ± 190	53 ± 2.1	96.9 ± 0.5
13	40HT—20ATIPA	0.044 ± 0.001	372 ± 161	434 ± 243	58 ± 2.1	99.0 ± 0.4
14	20ATIPA HDI	0.057 ± 0.001	293 ± 102	306 ± 115	45 ± 1.2	-
15	20ATIPA 50HDI/50TM	0.049 ± 0.002	517 ± 314	482 ± 287	50 ± 0.5	-
16	20ATIPA TM	0.049 ± 0.001	552 ± 214	488 ± 274	52 ± 0.5	-
17	25ATIPA 25HT 32 g	0.040 ± 0.001	515 ± 119	512 ± 249	-	-
18	30ATIPA 20HT 32 g	0.051 ± 0.005	415 ± 172	320 ± 137	-	-

Mean ± standard deviation presented for all data.
